# Complete genomes of *Halomonas*, *Pseudoalteromonas*, and *Brevibacterium* cheese rind bacteria

**DOI:** 10.1128/mra.00994-25

**Published:** 2026-03-11

**Authors:** Faith R. Hansen, Haley R. MacBurney, Abhishek Mohanty, Sofía I. Muñiz-Ferrer, Ena Stojanovic, Jolcey A. Santana, Alexa M. Newgard, Faith M. Rahic-Seggerman, Dylan L. Schultz, Lucille C. Jonas, Stephan Schmitz-Esser, Nick T. Peters

**Affiliations:** 1 Microbiology Undergraduate Program, Iowa State University 1177, Ames, Iowa, USA; 2 Department of Animal Science, Iowa State University 1177, Ames, Iowa, USA; 3 Department of Plant Pathology, Entomology, and Microbiology, Iowa State University 1177, Ames, Iowa, USA

**Keywords:** *Halomonas*, *Pseudoalteromonas*, *Brevibacterium*, cheese rind

## Abstract

Four different bacteria were isolated from cheese rinds, identified using genetic sequencing, and analyzed using various bioinformatic tools. The results provide perspective into the microbial diversity of cheese rinds, which contributes to our knowledge and understanding of cheese microbiomes and their role in food microbiology.

## ANNOUNCEMENT

Cheese rinds are complex microbial ecosystems that contribute significantly to aged cheeses’ flavor, texture, and aroma. Environmental and production factors like brining, ripening temperature and humidity, and washing techniques are crucial in determining which microbes thrive on cheese rinds (1, 2). Studying these microbes helps cheesemakers refine ripening practices and offers broader insight into microbial physiology in structured, competitive environments. Three alpine-style hard cheeses from Vermont and Wisconsin, USA, were used to isolate cheese rind bacteria and to sequence their genomes.

Bacterial strains were isolated and purified as previously described (3). Briefly, 1 g of cheese rind (see Table 1 for the different cheese types) was aseptically scraped off (avoiding the cheese core) and resuspended in sterile saline (0.9% NaCl) by vortexing. One hundred microliters of aliquots was serially diluted in 10-fold steps in sterile saline and plated on modified plate count agar plates (mPCA, containing 22.5 g/L PCA [Oxoid], skim milk 1 g/L, vancomycin 5 µg/mL; vancomycin was added to inhibit Gram-positive bacteria) and incubated under aerobic conditions at 30°C for 3–5 days. Individual colonies with different morphologies were picked, and DNA extraction was performed according to the DNeasy PowerLyzer PowerSoil Kit (Qiagen). PCR analysis of 16S rRNA genes was done using the Illustra/Cytiva PuReTaq Ready-To-Go Beads (Cytiva) using the primers 616F (5′-AGAGTTTGATCMTGGCTCAG-3′) and 1492R (5′- GTTACCTTGTTACGACTT-3′). NCBI BLASTn against the NCBI nonredundant (nr) database was used to identify the isolates. We isolated two *Halomonas* spp., one *Brevibacterium* sp., and one *Pseudoalteromonas* sp. For *Halomonas* KG2, *Pseudoalteromonas* KG3, and *Brevibacterium* LE-L, Illumina MiSeq and Oxford Nanopore Technologies (ONT) sequencing was performed at the Iowa State University (ISU) DNA facility. Illumina MiSeq library preparation was performed using the NEBNext Ultra II FS kit with standard parameters. ONT GridION sequencing was used for KG2, KG3, and LE-L; library preparation was performed using the SQK-LSK109 kit (no shearing of the DNA was performed) with barcoding kit EXP-NBD104 with standard parameters, sequencing was conducted on an X5 flow cell with Guppy 6.5.7 for base-calling. *Halomonas* H2 was sequenced by Plasmidsaurus, libraries were prepared with the ONT Rapid Barcoding Kit 96 V14 and sequenced on a PromethION P24 with an R10.4.1 flow cell, and the contigs were polished with Medaka (v1.11.1). Details for genome sequencing and assemblies can be found in Table 1. FastQC v0.11.9 was used to assess the quality of the reads (note: default parameters were used for all software unless specified otherwise). For Illumina sequencing, bases with quality scores of below 20 were trimmed and adapter sequences were removed using BBDuk v37.36. Bandage plots (v0.8.1) indicated complete circular genomes and plasmids. Genome-based taxonomic identification was done using the JSpecies webserver (4), using the Average Nucleotide Identity (ANI) ANIb, ANIm, and Tetra Correlation Search methods. KG2 had the highest ANI (around 95%) to various type strains in the genera *Halomonas* and *Vreelandella*. Isolate H2 showed lower similarity to different *Halomonas* type strains with ANI of approximately 83% (overlap 56%). KG3 had the highest similarity to *Pseudoalteromonas prydzensis* DSM 14232 (ANI 95%, overlap 72%), and LE-L had moderate similarity to different *Brevibacterium* type strains (ANI around 85%, overlap 65%). *Halomonas* H2 and *Brevibacterium* LE-L had ANI values below the species definition threshold (5); they might represent novel species, which requires further research for confirmation. Genomes were annotated with the BV-BRC server (6) and the NCBI-PGAP pipeline. Genome sequencing and assembly resulted in closed circular genomes and plasmids (if present) for all strains (Table 1). *Pseudoalteromonas* KG3 possessed two chromosomes, while all other strains had one chromosome. All strains had multiple plasmids (two to three) except for LE-L.

**TABLE 1:** Metadata, sequencing, and genomic characteristics of cheese rind bacteria

Isolate	H2	KG2	KG3	LE-L
Source and taxonomy of bacteria				
Taxonomy	*Halomonas* sp*.*	*Halomonas* sp.	*Pseudoalteromonas* sp.	*Brevibacterium* sp.
Isolation source	Alpine-style cheese (raw cow’s milk), VT, USA	Washed-rind, semi-soft cheese (pasteurized cow’s milk), VT, USA	Washed-rind, semi-soft cheese (pasteurized cow’s milk), VT, USA	Alpine-style cheese (raw cow’s milk), WI, USA
Genome sequencing				
Number of raw Illumina MiSeq reads		2,445,127	3,252,352	1,621,460
Illumina MiSeq read length (bp)		250	250	250
Total Illumina MiSeq sequencing data (Mbp)		1,222	1,626	486
Number of raw ONT reads	418,198	26,050	41,013	328,704
Average ONT read length (bp)	2,701	15,556	15,289	6,984.5
Total ONT sequencing data (Mbp)	1,129	405	627	2,295
Assembly				
Assembly software	Trycycler v0.5.4	Unicycler v0.4.8	Unicycler v0.4.8	Unicycler v0.4.8
Average Illumina MiSeq coverage		162×	239×	91×
Average ONT coverage	276×	85×	114×	125×
No. of contigs	3	4	5	1
Assembly N50 (bp)	3,973,152	4,461,952	3,931,114	4,194,401
Genome structure	One chromosome, two plasmids	One chromosome, three plasmids	Two chromosomes, three plasmids	One chromosome
Chromosome size (bp), [GC content]	3,973,152 [55.1%]	4,461,952 [52.9%]	3,931,114 [41.2%] (chromosome 1) 1,170,572 [40.7%] (chromosome 2)	4,194,401 [65.0%]
Plasmid size (bp), [GC content, coverage]	pHaH2_1: 88,701 [51.8%, 72×] pHaH2_1: 5,373 [55.2%, 9,445×]	pHaKG2_1: 98,093 [52.0%, 175×] pHaKG2_2: 4,719 [56.4%, 3,003×] pHaKG2_3: 4,213 [56.3%, 2,150×]	pPsKG3_1: 184,073 [39.5%, 263×] pPsKG3_2: 3,799 [39.6%, 2,708×] pPsKG3_3: 3,140 [40.8%, 3,360×]	
GenBank Accession number	CP189760 CP189761 CP189762	CP098528 CP098529 CP098530 CP098531	NZ_CP098527 NZ_CP098526 NZ_CP098525 NZ_CP098524 NZ_CP098523	CP189759
Illumina raw read accession number		SRX16175526	SRX16175528	SRX28630252
ONT raw read accession number	SRX28630250	SRX16175527	SRX16175529	SRX28630251

## Data Availability

The genome sequences have been deposited at NCBI under BioProject accession numbers PRJNA846065 (KG2, KG3) and PRJNA1254633 (H2, LE-L). GenBank and SRA accession numbers for each isolate are listed in Table 1.
